# Whispers of the polycystic ovary syndrome theater: Directing role of long noncoding RNAs

**DOI:** 10.1016/j.ncrna.2024.05.003

**Published:** 2024-05-14

**Authors:** Xiuying Lin, Xinyu Nie, Ping Deng, Luyao Wang, Cong Hu, Ningyi Jin

**Affiliations:** a Department of Pathology and Pathophysiology, Yan Bian University, Yanbian, Jilin, China; b Jilin Province People's Hospital, Changchun, Jilin, China; c Obstetrics and Gynecology Center, First Hospital of Jilin University, Changchun, Jilin, China; d Reproductive Medicine Center, Prenatal Diagnosis Center, First Hospital of Jilin University, Changchun, Jilin, China; e Medical Department, Jilin Provincial Cancer Hospital, Changchun, Jilin, China; f First Hospital of Jilin University, Changchun, Jilin, China; g Changchun Veterinary Research Institute, Chinese Academy of Agriculture Sciences Changchun, Jilin, China

**Keywords:** Polycystic ovary syndrome, Long noncoding RNAs, Granulosa cells, Hormone

## Abstract

Polycystic Ovary Syndrome (PCOS) is a multifaceted endocrine disorder that implicates a spectrum of clinical manifestations, including hormonal imbalance, metabolic dysfunction, and even compromised ovarian granulosa cell (GC) activity. The underlying molecular mechanisms of PCOS remain elusive, presenting a significant barrier to effective diagnosis and treatment. This review delves into the emerging role of long non-coding RNAs (lncRNAs) in the pathophysiology of PCOS, articulating their intricate interactions with mRNAs, microRNAs, and other epigenetic regulators that collectively influence the hormonal and metabolic milieu of PCOS. We examine the dynamic regulatory networks orchestrated by lncRNAs that impact GC function, steroidogenesis, insulin resistance, and inflammatory pathways. By integrating findings from recent studies, we illuminate the potential of lncRNAs as biomarkers for PCOS and highlight their contribution to the disorder, offering a detailed perspective on the lncRNA-mediated modulation of gene expression and pathogenic pathways. Understanding targeted lncRNA interactions with PCOS proposes novel avenues for therapeutic intervention to ameliorate the reproductive and metabolic disturbances characteristic of the syndrome.

## Abbreviations

(lncRNAs): Long noncoding RNAs
(PCOS): Polycystic ovary syndrome
(GCs): Granulosa cells
(IR): Insulin resistance
(LET): Low expression in tumor
(SRA): Steroid receptor RNA activator
(MALAT1): Metastasis-associated lung adenocarcinoma transcript 1
(ceRNA): competitive endogenous RNAs
(TGFβ): Transforming growth factor beta
(HOTAIR): lncRNA HOX transcript antisense RNA
(HDAC1): Histone deacetylase 1
(H3K4me3): Trimethylation of lysine 4 on histone H3
(PWRN2): Prader-Willi region non-protein coding RNA 2
(LH): Luteinizing hormone
(FSH): Follicle-stimulating hormone
(MEG3): Maternally expressed gene 3
(E_2_): Estradiol
(T): Testosterone
(AMH): Anti-Müllerian hormone
(BMI): Body mass index
(GLUT4): Glucose transporter type 4
(IL): Interleukin
(AUC): Area under the curve
(SNPs): Single-nucleotide polymorphisms

## 1 Introduction

Polycystic ovary syndrome (PCOS) is a complex endocrine disorder impacting approximately 5–20 % of females of reproductive age worldwide [1]. The Rotterdam Criteria, established in 2003 by the European Society for Human Reproduction and Embryology and the American Society for Reproductive Medicine, have not been definitively established [2]. PCOS is predominantly characterized by three hallmark symptoms: anovulatory infertility (resulting in irregular menses), hormonal disorders (such as hyperandrogenism), and polycystic ovarian morphology. Emerging investigations have focused on elucidating the role of ovarian granulosa cell (GC) dysfunction in the abnormal follicle development observed in patients with PCOS. Additionally, the occurrence of hyperandrogenemia can lead to a pronounced increase in the production of androgens, such as testosterone, further complicating the hormonal landscape. Insulin resistance (IR) is another contributing factor to the development of PCOS. PCOS is intricately linked to a heightened risk of obesity and a spectrum of metabolic abnormalities that extend its influence far beyond reproductive health, potentially leading to metabolic syndrome and cardiovascular disease. Despite extensive research, the exact mechanisms underlying the development of PCOS remain elusive.

To unravel the mysteries of PCOS etiology, recent studies dive deep into "omics" levels [[3], [4], [5]], which focus on RNA expression arrays, protein profiles, and methylation patterns. Long noncoding RNAs (lncRNAs) are an intriguing class of transcripts that extend beyond 200 nucleotides in length and are crucial components of the noncoding genome. LncRNAs do not participate in protein encoding; instead, they orchestrate complex biological mechanisms by regulating gene expression at multiple stages, including chromatin modification, transcription, and post-transcriptional processing. LncRNAs can regulate gene expression by directly interacting with mRNA transcripts and modulating their fate through various mechanisms. Some lncRNAs act as competing endogenous RNAs (ceRNAs), sequestering miRNAs and preventing them from binding and silencing target mRNAs. Other lncRNAs inhibit translation by interfering with the recruitment or assembly of the ribosome on the mRNA. Additionally, lncRNAs also promote mRNA decay by recruiting deadenylase complexes that remove the poly(A) tail or enzymes that cleave the 5’ cap, triggering exonucleolytic degradation. LncRNAs are pivotal regulators of cellular development, metabolism, and differentiation. Aberrant expression patterns have been associated with various diseases, including cancer, neurological disorders, and heart disease. The study of lncRNAs is akin to exploring new areas in the biosphere of genetics. Deep exploration of lncRNA profiles may potentially guide the identification of novel biomarkers capable of illuminating the path to early diagnosis and serve as targets for revolutionary therapies [6,7]. Importantly, further understanding of the aberrant expression of lncRNAs may provide novel insights into PCOS mechanisms, candidate biomarkers, and therapeutic targets.

In this review, we discuss the pivotal roles of lncRNAs in PCOS. Summarizing recent literature, we highlight substantial shifts in lncRNA profiles in PCOS. We aimed to elucidate the complex molecular mechanisms underlying PCOS and identify potential novel biomarkers that could transform its diagnosis. Furthermore, we discuss promising therapeutic strategies with the potential to improve the management of PCOS.

## 2 Regulation of GC function by lncRNAs in PCOS

PCOS is a complex endocrine disorder characterized by disrupted follicular maturation and ovulatory dysfunction, phenomena intimately linked to GC dysfunction. GCs are critical for folliculogenesis (maturation of ovarian follicles), steroidogenesis (production of steroid hormones), and regulation of oocyte maturation. Recent investigations using microarray technology have facilitated the comprehensive profiling of lncRNAs within ovarian GCs derived from patients with PCOS when compared with those of healthy controls [8]. LncRNAs derived from follicular fluid exosomes also play potential roles in GCs at the post-transcriptional level [9]. These screenings have revealed the differential expression of thousands of lncRNAs, suggesting a substantial role in the pathophysiology of PCOS [[10], [11], [12], [13]]. Understanding the molecular and cellular mechanisms that underlie GC dysfunction in PCOS is vital for developing targeted treatments that could address core aspects of this disorder.

### 2.1 Regulation of GC function by lncRNAs and messenger RNAs in PCOS

As an important function, lncRNAs interact with messenger RNAs, which carry the genetic code from DNA to the ribosome, the site for protein synthesis. Microarray analysis revealed that lnc-MAP3K13–7:1 was upregulated in luteinized GCs from both PCOS-affected and unaffected females. Lnc-MAP3K13–7:1 could directly interact with DNMT1 and trigger its degradation via ubiquitination, consequently inhibiting GC proliferation and contributing to the progression of PCOS [14]. Increased expression of the lncRNA low-expression in tumor (LET) reportedly decreases cell viability, impedes cellular migration, and terminates the epithelial-mesenchymal transition process while also promoting apoptosis in KGN cells, a human granulosa-like tumor cell line involved in steroidogenesis. This suppressive activity on cell dynamics was facilitated via the upregulation of tissue inhibitors of metalloproteinase 2 and the activation of both the Wnt/β-catenin and Notch signaling pathways [15]. In addition, the lncRNA steroid receptor RNA activator (SRA) was found to promote cell proliferation, alter the distribution of cell cycle phases, and suppress cell apoptosis by increasing the levels of BCL2 [16]. Suppression of lncRNA H19 inhibited GC proliferation by triggering apoptosis via targeting signal transducers and activators of transcription 3 signaling [17]. GCs of patients with PCOS exhibited elevated levels of LINC-01572:28 and p27 proteins, accompanied by a reduced amount of proliferating cell nuclear antigen protein. Upregulated LINC-01572:28 expression led to reduced cell proliferation and blockage of the G1/S phase transition of the cell cycle, which was partially mitigated by siRNA-mediated p27 knockdown [18]. In the nucleus of GCs, lncRNA metastasis-associated lung adenocarcinoma transcript 1 (MALAT1) was found to bind with MDM2, which binds to the 3’ end of MALAT1. Reduced MALAT1 expression resulted in elevated levels of p53 protein by decreasing its ubiquitination and subsequent degradation. Furthermore, MALAT1 facilitated the interaction between p53 and MDM2, thereby enhancing the proteasome-dependent breakdown of p53. Silencing MALAT1 in KGN cells and primary GCs increased apoptosis and decreased cell proliferation [19].

lncRNAs and mRNAs play crucial roles in the regulation of GC function in PCOS. These RNA molecules can influence a range of cellular processes, including cell proliferation, apoptosis, and transition through the cell cycle, which are vital for the proper functioning of GCs. Understanding the intricate network of lncRNAs and mRNAs and their impact on GCs can provide novel insights into potential therapeutic targets for the management of PCOS.

### 2.2 LncRNA- and microRNA (miRNA)-mediated regulation of GC function in PCOS

LncRNAs interact with miRNAs through multiple pathways and can profoundly impact miRNA-mediated regulation of gene expression, which plays a critical role in the pathogenesis, progression, and prognosis of diseases [20]. Key mechanisms include serving as molecular sponges, guiding miRNA processing or turnover, altering miRNA expression, modulating miRNA activity, and participating in feedback loops [21]. The identified ceRNA networks provide insights into facilitating PCOS diagnosis and treatment.

Recent studies have focused on the lncRNA “molecular sponges” that regulate GC proliferation and apoptosis and act as ceRNAs [22,23]. MALAT1, as a star molecule described earlier, was first confirmed to function as a ceRNA, interacting with two novel transforming growth factor beta (TGFβ) signaling negative regulators (miR-125b and miR-203a). Knockdown of MALAT1 in GCs induced the upregulation of miR-125b and miR-203a, thereby further repressed TGFβ/SMAD3 signaling, which resulted in upregulated cell proliferation and downregulated apoptosis [24]. Likewise, HAS2 antisense RNA 1 downregulated TGFβ signaling by inducing hypermethylation of the TGFBR2 promoter region, which aggravated the pathological processes in PCOS [25]. BBOX1-AS1 and CDKN2B-AS1 were found to be highly upregulated in PCOS and may serve as ceRNAs for miR-19b and miR-181a, respectively, to promote GC proliferation [26,27]. Furthermore, the lncRNA X-inactive specific transcript reportedly plays a critical role in PCOS by modulating the miR-30c-5p/BCL2L11 signaling axis and regulating the physiology of GCs [28]. LncRNA H19 also "sponges" miR-19b to regulate connective tissue growth factor expression in KGN cells, thereby promoting cell proliferation and reducing apoptosis [29]. Understanding the molecular sponge function of lncRNAs and its implications in health and disease is an active area of research, with the potential to uncover novel diagnostic and therapeutic avenues.

LncRNAs can act as decoys for miRNAs, binding and titrating them away from their mRNA targets, thereby relieving the mRNA from miRNA-mediated silencing. This decoy function of lncRNAs represents an important mechanism by which they can indirectly influence gene expression at both the transcriptional and post-transcriptional levels. For instance, lncRNA zinc finger antisense 1 can bind to miR-129 as a decoy to promote HMGB1 expression, thereby affecting endocrine disturbance, proliferation, and apoptosis of GCs in PCOS [30]. A direct interaction between lncRNA placenta-specific protein 2 and miR-19a has been identified. Overexpression of placenta-specific protein 2 in KGN cells led to upregulated tumor necrosis factor-α, which is also a target of miR-19a, which subsequently promoted KGN cell apoptosis [31]. In addition, through competitive binding with miR-130a, lncRNA HOX transcript antisense RNA (HOTAIR) increased insulin-like growth factor-1 expression and GC apoptosis, aggravating endocrine disorders in PCOS rat models [32]. Validation of sequencing findings revealed that HOTAIRM1 acts as a molecular sponge for miR-433-5p, thereby enhancing the expression of PIK3CD; this activity governs GC proliferation and apoptosis in PCOS [33]. Conversely, inhibition of miR-27a-3p could markedly counteract the insulin-like growth factor-1 reduction induced by HCP5 knockdown in GCs, which, in turn, enhanced cell viability and reduced apoptosis [34].

Moreover, other interactions between lncRNAs and miRNAs exert marked regulatory effects on gene expression. For example, PVT1 silencing was found to activate miR-17-5p/PTEN, leading to the inhibition of GC apoptosis and the promotion of PCOS proliferation [35]. LINC00477 targets GC apoptosis and proliferation via dysregulated candidate gene recombinant discs, large homolog associated protein 5, whereas miR-128 mimics could partially abrogate this effect [36,37]. Histone deacetylase 1 (HDAC1) reportedly participates in the etiology of PCOS. HDAC1 inhibited H3K9ac on the H19 promoter, and H19 competitively bound to miR-29a-3p to enhance NLRP3 expression. Overexpression of H19 or NLRP3, or inhibition of miR-29a-3p, could upregulate HDAC1 expression and promote GC pyroptosis [38]. HDAC1 and the trimethylation of lysine 4 on histone H3 (H3K4me3) are important posttranslational modifications that play critical roles in the regulation of gene expression. Crosstalk between these two modifications can occur through the recruitment of multi-protein complexes containing both histone deacetylases and methyltransferases, which can lead to the intricate control of gene expression. In rat models, exosomes from the follicular fluid increased LINC00092 expression, which suppressed PTEN transcriptional activity by associating with KDM5A and promoting the demethylation of H3K4me3; this, in turn, reduced apoptosis in ovarian cells and mitigated PCOS-related symptoms [39]. Analysis of three microarray datasets on oocyte development in patients with PCOS identified the presence of lncRNA Prader-Willi region non-protein coding RNA 2 (PWRN2), miR-92b-3p, and TMEM120B in cumulus cells at varying stages of maturity. PWRN2 was confirmed to be crucial for the nuclear maturation of oocytes in PCOS, functioning as a ceRNA to sequester miR-92b-3p, thus preserving TMEM120B during oocyte maturation [40]. Furthermore, PWRN2 may bind to LSD1 to inhibit ATRX transcription, thereby restraining the growth of GCs and promoting PCOS progression [41]. Moreover, lncRNAs can bind to the Drosha or Dicer complexes, which are responsible for miRNA maturation, thereby influencing the processing and maturation of specific miRNAs [42]. LncRNAs can also bind directly to RNA-binding proteins, preventing them from interacting with their usual mRNAs or pre-mRNA targets, potentially altering mRNA splicing, stability, or translation. By binding to the RNA-binding protein LIN28B, LINC00092 not only inhibits the biogenesis of pre-miR-18a-5p but also enhances miR-18b-5p expression, thereby suppressing the PTEN signaling pathway to enhance cell viability and migration [43]. In addition, TMPO-AS1 was found to be localized in both the nucleus and cytoplasm. TMPO-AS1 overexpression not only decreased mature miR-355-5p levels but also increased premature miR-355-5p levels, highlighting the dichotomous participation of this lncRNA in KGN cell proliferation [44]. miRNAs are not invariably function downstream of lncRNAs, such as, miR-21 can downregulate SNHG7 expression, prevent KGN cell proliferation, and induce GC apoptosis [45].

**Fig. 1:**
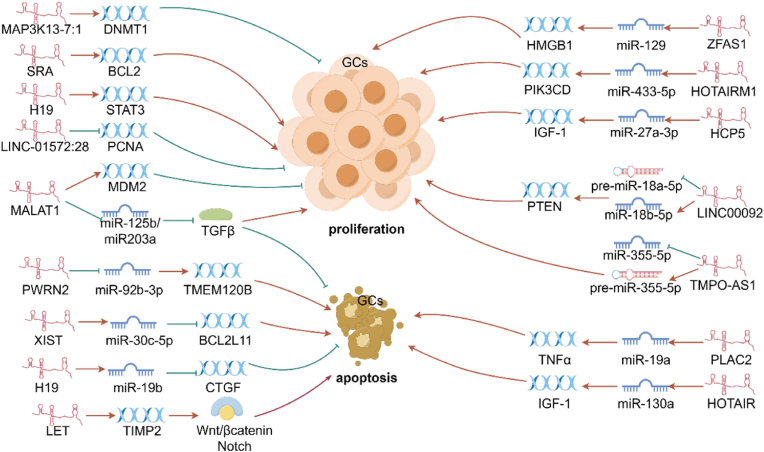
**Schematic diagram: The impact of lncRNAs on ovarian GCs activity in PCOS at both transcriptional and post-translational levels.** The figure illustrates the multifaceted roles of lncRNAs in modulating the activity of ovarian GCs in PCOS. Lnc-MAP3K13–7:1 induces DNMT1 degradation, reducing GC proliferation. SRA promotes GC proliferation and inhibits apoptosis by elevating BCL2 levels. H19 suppression curtails GC proliferation by promoting apoptosis through STAT3 signaling. Elevated LINC-01572:28 reduced PCNA characterize PCOS GCs. MALAT1 silencing increases apoptosis and decreases proliferation by interacting with MDM2 and modulating miR-125b and miR-203a, which suppress TGFβ signaling. PWRN2 sequesters miR-92b-3p to preserve TMEM120B, aiding oocyte maturation. XIST impacts PCOS by regulating the miR-30c-5p/BCL2L11 axis. H19 “sponges” miR-19b to modulate CTGF expression, promoting GC proliferation and survival. LET enhances apoptosis via TIMP2 upregulation and activation of Wnt/β-catenin and Notch pathways. ZFAS1 binds miR-129 to enhance HMGB1 expression, affecting GC dynamics. HOTAIRM1 sponges miR-433-5p, upregulating PIK3CD. miR-27a-3p inhibition opposes IGF-1 reduction by HCP5 knockdown, improving cell viability and reducing apoptosis. LINC00092 suppresses pre-miR-18a-5p biogenesis and upregulates miR-18b-5p, inhibiting the PTEN pathway to bolster cell viability and migration. TMPO-AS1 modulates miR-355-5p levels, impacting GCs proliferation. Overexpression of the lncRNA PLAC2 in KGN cells upregulated TNF-α/miR-19a, leading to increased KGN cell apoptosis. HOTAIR competes with miR-130a to increase IGF-1 levels and GC apoptosis.

In PCOS, lncRNAs and miRNAs play pivotal roles in regulating GC function, which is essential for normal ovarian function and fertility (Fig. 1). LncRNAs can function as ceRNAs by sequestering specific miRNAs to prevent them from inhibiting their target mRNAs, thereby influencing the pathophysiology of PCOS. Comprehensively elucidating these intricate molecular interactions can offer valuable insights into the potential mechanisms underlying PCOS and may lead to the identification of novel therapeutic targets for this complex endocrine disorder.

## 3 LncRNAs and hormone dysregulation in PCOS

Polycystic morphology is typically attributed to the arrest of antral follicle development, leading to the accumulation of small follicles that fail to attain full maturity. Disrupted follicular genesis in PCOS is believed to be associated with intrinsic abnormalities in GCs, which play a pivotal role in follicle development and hormone production. GCs are responsible for converting androgens to estrogens via aromatase, and this conversion is impaired in PCOS. In patients with PCOS, GCs frequently exhibit altered responsiveness to luteinizing hormone (LH) and follicle-stimulating hormone (FSH), which further exacerbates dysfunction in follicle development and ovulation [46].

Silencing of lncRNA maternally expressed gene 3 (MEG3) attenuated sex hormone dysregulation and ovarian histopathological changes in rats with PCOS and promoted follicle cell development and maturation by targeting the miR-21-3p/PI3K/AKT/mTOR pathway. Meanwhile, acupuncture intervention suppressed GC apoptosis in rats with PCOS by targeting the lncMEG3/miR-21-3p axis [47,48]. Furthermore, silencing of MALAT1 and MEG3 inhibited testosterone release and attenuated sex hormone dysregulation [49]. Conversely, MALAT1 also exerted beneficial effects against ovarian tissue injury and endocrine disorders in rats with PCOS. Overexpression of MALAT1 not only reduced levels of FSH and elevated serum levels of estradiol (E_2_), testosterone, and LH but also promoted the expression of the interleukin (IL)-6 family cytokine (LIF), which could be reversed by overexpression of miR-302d-3p [50]. In an *in vivo* experiment, lentivirus-mediated knockdown of lncRNA urothelial carcinoma associated 1 in the ovaries of PCOS mice attenuated ovarian structural damage [51]. Additionally, lncRNA nuclear-enriched abundant tran 1 reportedly functions as a ceRNA to adsorb miR-381 and target IGF1, effectively improving sex hormone levels in rats with PCOS [52,53]. The novel lncRNA CTBP1-AS, which modulates androgen receptors, has been linked to PCOS and serves as an indicator of serum T-level variations in Chinese patients with PCOS [54]. H19 deficiency could interfere with androgen synthesis via a mechanism involving Cyp17, the crucial enzyme for androgen production in the gonads and adrenal cortex. Conversely, an abundance of H19 may contribute to the development of high androgen levels associated with PCOS [55]. In PCOS, lncRNA BANCR was found to enhance cell apoptosis by increasing Bax and p53 levels [56]. Overexpression of lncRNA SRA was shown to enhance levels of E_2_ and progesterone, as well as increase the expression of their essential enzymes, cytochrome P450 family 11 subfamily A member (CYP19A1) and CYP11A1 [16]. In PCOS model mice, knockdown of the lncRNA SRA affected insulin secretion, decreased ovarian damage, and reduced levels of angiogenic factors. Furthermore, shRNA against lncRNA SRA suppressed the production of proinflammatory cytokines (such as TNF-α, IL-1β, and IL-6), as well as the translocation of NF-κB into the nucleus in both PCOS mouse ovaries and primary GCs treated with dehydroepiandrosterone sulfate (DHEA) [57]. In rodent models of PCOS, si-HOTAIR treatment lowered serum T, E_2_, and LH levels, increased FSH levels, normalized estrous cycles and ovarian structure, and reduced ovarian cell apoptosis. In cell studies, si-HOTAIR improved GC survival while reducing GC apoptosis. Moreover, si-HOTAIR increased Bcl2 expression, decreased Bax, Bad, and IGF1 expression levels, and downregulated the activity of the PI3K/AKT pathway. These *in vitro* effects were amplified by LY294002 and were partially negated by IGF1 [58].

**Fig. 2:**
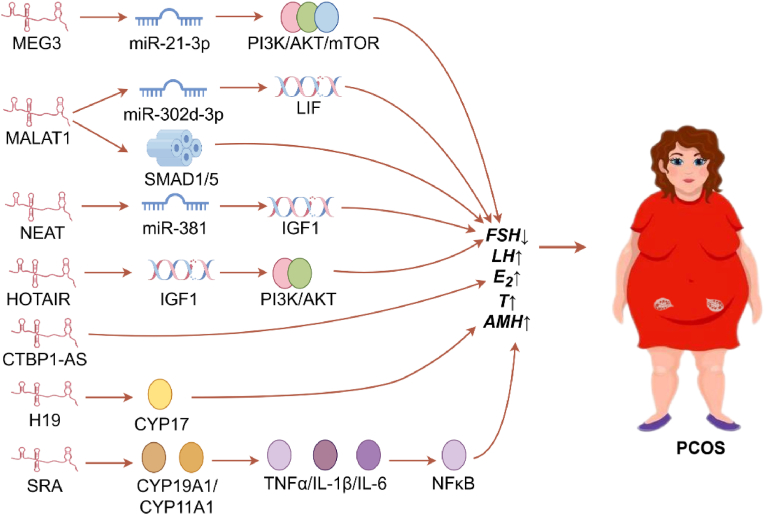
The Impact of lncRNAs on hormone dysregulation and GCs function in PCOS. The figure summarizes the regulatory roles of various lncRNAs in PCOS. Lnc MEG3 silencing reduces miR-21-3p, modulating the PI3K/AKT/mTOR pathway, thus normalizing sex hormone levels and improving follicle development. Overexpression of the lnc MALAT1 disrupted the hormonal balance by decreasing FSH, increasing E_2_, T and LH levels, and promoting LIF expression, which was reversed by miR-302d-3p, while AMH stimulation upregulated MALAT1 and SMAD1/5 phosphorylation in KGN cells. NEAT1 elevates IGF1 by sponging miR-381, improving sex hormone levels. HOTAIR regulates serum hormone levels, estrous cycles, and GCs apoptosis, influencing the PI3K/AKT pathway. CTBP1-AS is associated with serum testosterone level variations. H19 plays a role in androgen synthesis and involves modulating Cyp17. Overexpressed SRA lncRNA increases E_2_ and progesterone, while its knockdown reduces inflammatory markers and insulin secretion, mitigating ovarian damage.

GCs are involved in the production of the anti-Müllerian hormone (AMH), which is typically elevated in patients with PCOS and is thought to play a role in follicular arrest observed associated with this condition. Elevated AMH levels may contribute to the decreased sensitivity of follicles to FSH, thereby promoting a state of anovulation. External AMH stimulation increased MALAT1 levels and phosphorylation of SMAD 1/5 (Ser463/465) proteins, suggesting that AMH may regulate MALAT1 expression in KGN cells [59]. In rats with PCOS, acupuncture intervention was found to suppress autophagy and GC proliferation, as well as decrease lncMEG3 expression, consequently inhibiting the PI3K/AKT/mTOR pathway. This contributed to rectifying the abnormal follicular development, substantially lowering serum levels of LH, FSH, testosterone, and AMH, and notably enhancing E_2_ levels [12]. LINC-01572:28 expression was positively correlated with baseline testosterone levels [18] (shown in Fig. 2).

## 4 LncRNAs and metabolic disturbances in PCOS

The primary metabolic disturbances in PCOS include IR and hyperinsulinemia. When fat cells fail to respond to insulin effectively, the pancreas produce more insulin as a compensatory mechanism, leading to hyperinsulinemia. These hormonal imbalances can further disrupt normal lipid metabolism in the body, leading to dyslipidemia. High insulin levels exacerbate the ovarian production of androgens, contributing to the hormonal imbalances characteristic of PCOS, such as elevated testosterone levels. The interplay between IR and elevated androgen levels can also lead to weight gain and obesity, particularly central obesity, which further aggravates IR and creates a vicious cycle.

In KGN cells, the knockdown of lncRNA OC1 reduced the cell viability, enhanced apoptosis, and increased aromatase mRNA levels and estradiol production. In a murine PCOS model, lnc OC1 stimulated insulin release, the production of angiogenic factors, and IκBα phosphorylation, effects that were partly reversed by lnc-OC1 shRNA [60]. Elevated levels of H19 markedly improved the effectiveness of the combined sitagliptin and metformin treatment in a rat model of PCOS induced by insulin and human chorionic gonadotropin. Additionally, combined sitagliptin and metformin therapy upregulated H19 expression by inhibiting the PI3K/AKT-DNMT1 pathway, which, in turn, reduced apoptosis and IR in PCOS model cells and ameliorated hormonal imbalances, ovarian polycystic alterations, and IR in a PCOS rat model [61]. Furthermore, metformin was found to suppress H19 expression by activating the miR-29b-3p/AMPK signaling pathway in cell or serum samples collected from PCOS rat models [62]. Obese individuals with PCOS have a higher risk of developing endometrial hyperplasia, a condition closely linked to the expression of insulin-responsive glucose transporter type 4 (GLUT4). Metformin reportedly exerts therapeutic benefits in endometrial hyperplasia by regulating the MEG3/miR-223/GLUT4 and SNHG20/miR-4486/GLUT4 signaling pathways [61]. Overexpression of CCNL in GCs increased FOXO1 expression, which, in turn, enhanced apoptosis, diminished the glucose transport ability, and disrupted mitochondrial function; however, these effects were partially reversed by silencing FOXO1. Therefore, CCNL may contribute to the progression of PCOS, potentially playing a role in disease manifestations, such as follicular atresia and IR [63]. Nevertheless, in addition to their persistently negative role in metabolic disturbances in PCOS, lncRNAs may positively impact PCOS. For example, lncRNA growth-arrest-specific transcript 5 has been identified as a protective factor. Decreased serum growth-arrest-specific transcript 5 levels may play a role in exacerbating IR and elevating IL-18 secretion in patients with PCOS [64].

These interactions have important implications in the pathophysiology of PCOS, where alterations in GC behavior contribute to hormonal imbalances, metabolic dysfunctions, and fertility-related challenges characteristic of the condition**.**

## 5 LncRNAs as predictors in PCOS

The potential use of lncRNAs as disease predictors can be attributed to several characteristics, particularly their tissue specificity. Understanding the role of specific lncRNAs in disease pathogenesis can provide insights into the molecular mechanisms driving the disease and potentially lead to the discovery of new therapeutic targets.

In a previous study, the researchers enrolled 65 PCOS patients and 65 healthy control individuals. From this participant pool, they selected 6 GC samples from each group and subjected them to high-throughput sequencing analysis. Through this approach, the researchers identified a set of seven lncRNAs and three mRNAs that were abnormally expressed in individuals with PCOS compared to the healthy controls. These lncRNAs showed promise as diagnostic markers, with receiver operating characteristic and area under the curve (AUC) values of 0.6807 and 0.6410, respectively. These AUC values were pivotal in differentiating whether the rate of high-quality embryos exceeded 50 % [65]. Serum lncRNA profiles were found to differ between non-obese patients with PCOS and control subjects, and this expression pattern varied from those observed in obese patients with PCOS from the same ethnic group; however, these differences were not associated with AMH, androgens, or IR [66,67]. LncRNA SRA1 was substantially overexpressed in females with PCOS when compared with that in control subjects, with a positive correlation noted between lncRNA SRA1 expression and BMI in the PCOS group [68]. Although single-nucleotide polymorphisms (SNPs) within the regulatory regions of lncRNAs can impact their expression, Sanger sequencing revealed no significant link between the two SNPs in the lncRNA SRA1 gene (rs801460 and rs250426) and PCOS susceptibility (*p* > 0.05). However, the testosterone allele at SNP rs10463297 in SRA1 was associated with a lower risk of PCOS than the C allele (OR = 0.63, 95%CI: 0.50–0.79, *p* < 0.01). This association was particularly significant among individuals with a BMI ≥26.5 kg/m^2^ carrying the TC and CC genotypes when compared with those in the TT genotype (OR = 0.54, 95%CI: 0.33–0.89, *p* = 0.02). Across different age and BMI categories, rs10463297 was markedly associated with susceptibility to PCOS. According to multifactor dimensionality reduction analysis, a combination of factors, including age, BMI, and SNPs rs801460, rs10463297, and rs250426, constituted a “high-risk combination” for PCOS, with a susceptibility risk nearly six times that of the “low-risk combination” (95%CI: 4.14–8.56, *p* < 0.01). The TCT haplotype constructed from SRA1 SNPs was linked to an increased risk of PCOS, whereas the CTT haplotype was associated with a reduced risk (OR = 1.66, 95%CI: 1.20–2.30, *p* < 0.01). SNP rs10463297 also correlated with peripheral blood leukocyte levels of lncRNA SRA1, which, in turn, has been linked to PCOS susceptibility [69]. Moreover, patients with PCOS exhibited substantially higher levels of lncRNA H19 than control subjects, and the elevated levels positively correlated with fasting plasma glucose levels in the PCOS group; however, lncRNA H19 showed no significant relation with total T or IR in either group [70]. After adjusting for age and body mass index (BMI), patients with high lncRNA ENST00000550337.1 expression were found to be substantially more prone to developing PCOS than those with normal expression. Expression levels predicted PCOS with a sensitivity of 81.3 % and a specificity of 78.1 % [71]. Notably, the H19 rs2067051G > A variant was found to be associated with an increased risk of PCOS, observed in a study that included 115 PCOS patients and 130 healthy control individuals [72]. Downregulation of another lncRNA, lncRNA X-inactive specific transcript, may be involved in the development of PCOS and was associated with adverse pregnancy outcomes in affected individuals [73]. Compared with the control group, patients with PCOS showed upregulated expression of lncRNA ROR and downregulated miR-206 expression. Based on logistic regression analysis, lncROR and miR-206 could be independent predictors of PCOS. The receiver operating characteristic curve demonstrated that lncROR could be a diagnostic marker for PCOS, with an AUC of 0.893. Inhibition of the lncROR/miR-206/vascular endothelial growth factor pathway led to decreased cell proliferation and increased cell apoptosis [74]. Most discussed lncRNAs, which are associated with GC function and hormone dysregulation, are also overexpressed in patients with PCOS rather when compared with that in controls, and further research is needed to verify the predictor characteristics.

As research continues to unravel lncRNAs functions, these molecules are likely to become more integrated into clinical practice for diagnosis, prognosis, and personalized therapy. However, the exact mechanisms are not fully understood. Many lncRNAs exhibit tissue-specific expression patterns, and obtaining samples from specific tissues can be challenging in clinical settings. Demonstrating the clinical utility of lncRNAs as predictors and therapeutic targets in PCOS will require larger, well-designed studies to validate their diagnostic and prognostic value, as well as the efficacy of lncRNA-based therapies. Although the potential of lncRNAs as disease predictors is significant, there are challenges in their clinical implementation.

## 6 Others

In addition to promoting GC proliferation and apoptosis, interactions between lncRNAs and GCs, ferroptosis, autophagy, epithelial-mesenchymal transition, and other biological processes are associated with PCOS pathogenesis [75].

Patients with PCOS frequently experience a concurrent state of chronic low-grade inflammation, which may contribute to PCOS by promoting immune imbalance [76]. Overexpression of AOC4P was found to promote KGN cell proliferation and suppress apoptosis. After co-culture with AOC4P overexpressed KGN cells, the immune balance was prone to an anti-inflammatory profile, characterized by elevated M2/M1 macrophages, T helper cell type 1/T helper cell type 2 ratio, and regulatory T cell counts. Finally, overexpression of AOC4P blocked the nuclear factor κB pathway in KGN cells [77]. Increased IL-6 levels have also been associated with the development of PCOS. Overexpression of lncRNA SRLR led to upregulated IL-6 expression and promoted the apoptosis of human KGN cells [78]. Table 1 summarizes lncRNAs associated with PCOS.

**Table 1:** lncRNAs associated with PCOS.

Sample	LncRNAs	Expression in PCOS	Influence and mechanism	Reference
KGN cells	AOC4P (amine oxidase copper containing 4, pseudogene)	Down	Overexpression of AOC4P promoted KGN cell proliferation and inhibited apoptosis. After co-culture with AOC4P overexpressed KGN cells, subtype of macrophages and T cells shift. Finally, it inhibited the activation of the NF-κB pathway.	[77]
Human GCs, KGN cells	BANCR (BRAF- activated non-protein coding RNA)	Up	lncRNA BANCR participates in PCOS by promoting cell apoptosis through the upregulation of Bax and p53.	[56]
Human follicular fluid	BBOX1-AS1 (BBOX1 antisense RNA1)	Up	BBOX1-AS1 is highly upregulated in PCOS, and it may serve as an ceRNA for miR-19b to suppress its role in inhibiting GCs proliferation.	[26]
Human GCs	CCNL (CCNL1-3:1)	Up	CCNL overexpression upregulated FOXO1 expression, promoted cell apoptosis, reduced glucose transport capability, and impaired mitochondrial function, and these effects were partially abolished by silencing FOXO1.	[63]
Rat endometrium	CD36-005	Up	A total of fifty-five mRNAs differentially expressed were identified in CD36-005 overexpressed stromal cells.	[5]
Human GCs	CDKN2B-AS1(Cyclin dependent kinase inhibitor 2B-AS1)	Up	CDKN2B-AS1 is overexpressed in PCOS and may sponge miR-181a to promote granulosa cell proliferation.	[27]
Human serum	CTBP1-AS (C-Terminal binding protein 1 antisense)	Up	Abnormal CTBP1-AS expression is a risk factor for PCOS and it is a predictor of variability in serum total testosterone level in Chinese women with PCOS.	[54]
Human serum	ENST00000550337.1	Up	Patients with elevated lncRNA ENST00000550337.1 expression had significantly increased PCOS risk after adjusting for age and BMI. LncRNA ENST00000550337.1 expression level provided a sensitivity of 81.3 % and a specificity of 78.1 % to predict PCOS.	[71]
Human serum	GAS5(Growth-arrest specific transcript 5)	Down	Elevated IL-18 expression and downregulation of GAS5 in serums might contribute to IR in PCOS patients.	[64]
KGN cells	GAS5 (growth arrest specific 5)	Up	Overexpression of GAS5 led to upregulated IL-6, and significantly decreased apoptosis rate.	[64]
KGN cells, HGL5 cells and mice GCs, KGN cells, Human and mice GCs Human serum Rat GCs Rat serum and GCs Human blood	H19	Up	Suppression of H19 caused the inhibition of GCs proliferation due to the triggering of apoptosis. Silencing of STAT3 suppressed the expression of H19 in GCs and also halted their growth by triggering apoptosis. STAT3 and H19 silencing cause inhibition of GCs growth synergistically. HDAC1 inhibited H3K9ac on the H19 promoter and H19 competitively bound to miR-29a-3p to improve NLRP3 expression. Overexpressed H19 or NLRP3 or inhibited miR-29a-3p reversed the inhibition of GCs pyroptosis by HDAC1 upregulation. H19 acted as a ceRNA to bind to miR-19b via a "sponge" to regulate the effect of connective tissue growth factor (CTGF) on GCs, then promote cell proliferation and decrease cell apoptosis. Loss of H19 may disrupt androgen production via a Cyp17 (the rate-limiting enzyme for the formation of androgens in the gonads and adrenal cortex)-mediated mechanism. Upregulation of H19 improved reproductive hormone disorders, ovarian polycystic changes, and IR of PCOS rats via suppressing the PI3K/AKT-DNMT1 pathway. The administration of MET suppressed the expression of H19 while elevating the expression of miR-29b-3p through AMPK signaling pathways in cell or serum samples collected from PCOS rats. MET exhibits a therapeutic effect in the treatment of PCOS by reducing the expression of MMPs. H19 rs2067051G > A is associated with an increased risk of PCOS in the Iranian population.	[17] [38] [29] [55] [61] [62] [70]
Human GCs and KGN cells	HAS2-AS1 (HAS2 antisense RNA 1)	Up	Upregulation of HAS2-AS1 promoted GCs migration, proliferation and repressed apoptosis. HAS2-AS1 overexpression induced H3K27 hypermethylation in the TGFBR2 promoter region and then repressed TGFBR2 transcription in GCs.	[25]
KGN cells	HCP5 (*human histocompatibility leukocyte antigen complex p5*)	/	miR-27a-3p was a directly target to HCP5 and it could directly bind with insulin-like growth factor-1 (IGF-1). HCP5 might be a potential regulatory factor for development of PCOS through regulating the miR-27a-3p/IGF-1 axis to block the effects on the proliferation and apoptosis in GCs.	[34]
Human serum and GCs	HLA-F-AS1 (HLA-F antisense RNA1)	down	HLA-F-AS1 increased ovarian granulosa cell proliferation and inhibited cell apoptosis. miR-613 played an opposite role and suppressed the role of HLA-F-AS1.	[42]
Human and rat GCs	HOTAIR (HOX transcript antisense RNA)	Up	LncRNA HOTAIR up-regulates the expression of IGF-1-mediated PI3K/Akt pathway and aggravates the endocrine disorders and GCs apoptosis through competitive binding to miR-130a in rat models of PCOS.	[32,58]
Human GCs, KGN cells	HOTAIRM1 (HOXA transcript antisense RNA, myeloid-specific 1)	Up	HOTAIRM1 could sponge miR-433-5p to promote PIK3CD expression, thereby regulating the growth and apoptosis of granulose cells in PCOS.	[33]
KGN cells	LET (low expression in tumor)	/	Overexpression of LET inhibits cell viability, migration and EMT process, and increases apoptosis in GCs by up-regulating the expression of TIMP2 and activating the Wnt/β-catenin and notch signaling pathways.	[15]
KGN cells Rat follicular fluid	LINC00092	/	Overexpression of LINC00092 significantly reduces apoptosis in GCs while enhancing cell viability and migration. By binding to the RNA-binding protein LIN28B, LINC00092 not only inhibits the biogenesis of pre-miR-18a-5p but also increases the expression of miR-18b-5p, thereby suppressing the PTEN signaling pathway. Follicular fluid-derived exosomes augmented LINC00092 expression in rats. LINC00092 bound to lysine demethylase 5 A (KDM5A), and KDM5A facilitated the demethylation of H3K4me3 to restrain the transcriptional activity of PTEN, thereby reducing apoptosis in ovarian cells and alleviating PCOS symptoms.	[39] [43]
Human and mice serum, Human GCs	LINC00477	Up	LINC00477 overexpression inhibited the proliferation and promoted the apoptosis of granulosa cells. Knockdown DLGPA5 (downstream of LINC0047) repressed viability and proliferation, but enhanced apoptosis and disrupted cell cycle in GCs.	[36]
Human GCs	LINC-01572:28	Up	LINC-01572:28 suppresses cell proliferation and cell cycle progression by reducing the degradation of p27 protein via SKP2.	[18]
Human GCs and serum Rat serum and GCs Human GCs and KGN cells Human GCs and KGN cells	MALAT1 (metastasis-associated lung adenocarcinoma transcript 1)	Up Down	MALAT1 and phosphorylated SMAD 1/5 (Ser463/465) protein were upregulated by AMH stimulation, MALAT1 inhibit granulosa cell proliferation and may be correlated with pregnancy outcome in PCOS. MALAT1 reduction in GCs regulate TGFβ signaling through sponging miR-125b and miR-203a, which may contribute to the pathophysiological processes of PCOS. Overexpression of MALAT1 reduced endocrine disorders and ovarian tissue damage via the miR-302d-3p/LIF axis. Knockdown of MALAT1 in GCs increased p53 protein levels by repressing p53 ubiquitination and degradation, increased apoptosis and reduced proliferation.	[59] [24] [49] [19]
Human GCs and KGN cells	MAP3K13–7:1	Up	Lnc-MAP3K13-7:1-dependent DNMT1 inhibition regulates CDKN1A/p21 expression and inhibits GCs proliferation.	[14]
Human and rat GCs	MEG3 (maternally expressed gene 3)	Up	Silencing of MEG3 attenuated sex hormone dysregulation and ovarian histopathological changes in PCOS rats and promoted follicle cell development and maturation by targeting miR-21-3p/PI3K/AKT/mTOR pathway.	[12,47]
Human and rat GCs, Human and mice GCs	NEAT1 (nuclear-enriched abundant tran 1)	Up	NEAT1 acted as a ceRNA to adsorb miR-381 to target IGF1, effectively improving the levels of sex hormones and reducing the pathological damage of ovarian tissue in rats with PCOS. NEAT1 exacerbates metabolic disorders and ovarian pathological changes in PCOS mice by downregulating miR-324-3p and upregulating BRD3.	[49] [52]
Human and mice GCs, KGN cells	OC1(ovarian cancer associated 1)	Up	In PCOS mice, Lnc-OC1 promoted the serum insulin release, production of angiogenesis-related factors and IκBα phosphorylation, which could be partially restored by Lnc-OC1 shRNA.	[60]
Human GCs	PLAC2 (placenta-specific protein 2)	Up	Overexpression of PLAC2 led to upregulated TNF-α, which is a target of miR-19a. PLAC2 and TNF-α promoted the apoptosis of GCs.	[31]
Human GCs and follicular fluid	PVT1 (plasmacytoma Variant Translocation 1)	Up	Inhibited lncRNA PVT1 and overexpressed miR-17-5p result in downregulation of PTEN and promotion of cell proliferation, as well as inhibition of apoptosis of ovarian granulosa cells in PCOS.	[35]
Human cumulus cells KGN cells	PWRN2 (Prader-Willi region nonprotein coding RNA 2)	Up	PWNR2 plays important roles in oocyte nuclear maturation in PCOS by functioning as a ceRNA to reduce the availability of miR-92b-3p for TMEM120B target binding during oocyte maturation in PCOS. PWRN2 might restrain GCs growth to promote PCOS progression, which was achieved by binding with LSD1 to inhibit ATRX transcription.	[40] [41]
KGN cells	ROR (regulator of reprogramming)	Up	Down regulation of lncROR/miR-206/VEGF pathway inhibited cell proliferation and promoted cell apoptosis.	[74]
Human ovarian, KGN cells	SNHG7(small nucleolar RNA host gene 7)	/	miR-21 downregulated SNHG7, then the proliferation of KGN cells was prevented and apoptosis was induced in GCs.	[45]
Endometrium of PCOS rats	SNHG20 (small nucleolar RNA host gene 20)	Up	Metformin exhibited a therapeutic effect in the treatment of EH by modulating the lncRNA-MEG3/miR-223/GLUT4 and lncRNA-SNHG20/miR-4486/GLUT4 signaling pathways. This work provides mechanistic insight into the development of EH.	[48]
Mice GCs Human PBMCs	SRA (steroid receptor RNA activator)	Up	Overexpression of SRA stimulated GCs cell growth, changed distribution of cell cycle phases, and inhibited cell apoptosis with up-regulation of bcl2 and down-regulation of bax, cleaved-caspase 3, and cleaved-PARP. Moreover, the contents of estradiol and progesterone were up-regulated following over-expression of SRA. rs10463297 SNP was correlated with the level of lncRNA SRA1 in the peripheral blood leukocytes, which is associated with PCOS susceptibility.	[16,68] [69]
KGN cells	SRLR (sorafenib resistance in renal cell carcinoma associated)	Up	Overexpression of the SRLR led to upregulation of IL-6 and promoted apoptosis of GCs.	[78]
Human follicular fluid, KGN cells	TMPO-AS1 (TMPO antisense RNA 1)	/	TMPO-AS1 overexpression decreased mature miR-355-5p level but increased premature miR-355-5p. TMPO-AS1 was localized in both nucleus and cytoplasm. TMPO-AS1 increased KGN cell proliferation while miR-355-5p decreased cell proliferation. TMPO-AS1 reduced the suppressive effects of miR-355-5p on cell proliferation.	[44]
Mice GCs and serum	UCA1 (urothelial carcinoma-associated 1)	Up	UCA1 silencing attenuated the ovary structural damage, increased the number of granular cells, inhibited serum insulin and testosterone release, and reduced the pro-inflammatory cytokine production, through PI3K/AKT signaling pathway regulation.	[51]
KGN cells	XIST (X-inactive specific transcript)	Up	Overexpression of XIST decreased miR-30c-5p then increased BCL2L11 expression, inhibited GCs proliferation, and induced apoptosis. Downregulation of Xist expression may be involved in PCOS and is correlated with adverse pregnant outcomes in PCOS.	[28,73]
Human GCs	ZFAS1(zinc fnger antisense 1)	Up	ZFAS1 could bind to miR-129 to promote HMGB1 expression, thereby affecting the endocrine disturbance, proliferation and apoptosis of ovarian granulosa cells in PCOS.	[30]

## 7 Conclusions

The etiopathogenesis of PCOS is multifactorial and involves dysregulated steroidogenesis, impaired GC proliferation, and anomalous oocyte development. Accumulated evidence has identified most lncRNAs with upregulated expression in the PCOS cohort. In this review, we summarized the complex interplay between lncRNAs and the function of GCs, along with the contributions of lncRNAs to the delicate equilibrium of hormone levels and their intricate relations with glycolipid metabolism. We further highlighted the potential role of lncRNAs as molecular sentinels and therapeutic targets. Envisioning the future of PCOS management, we anticipate a paradigm shift toward lncRNA-centric diagnostics and personalized interventions, potentially revolutionizing treatment strategies and improving prognostic outcomes of patients with PCOS worldwide.

## Funding

10.13039/501100011789Science and Technology Department of Jilin Province (YDZJ202201ZYTS085, 20220204020YY, 20230204036YY, 20230203051SF, YDZJ202301ZYTS113, YDZJ202301ZYTS119), 10.13039/501100001809National Natural Science Foundation of China (82201847). Tianhua Health Public Welfare Foundation of Jilin (J2022JKJ026, J2023JKJ025).

## Data availability

No data was used for the research described in the article.

## CRediT authorship contribution statement

**Xiuying Lin:** Writing – original draft. **Xinyu Nie:** Writing – original draft. **Ping Deng:** Writing – original draft. **Luyao Wang:** Writing – original draft. **Cong Hu:** Writing – review & editing. **Ningyi Jin:** Writing – review & editing.

## Declaration of competing interest

The authors declare that they have no known competing financial interests or personal relationships that could have appeared to influence the work reported in this paper.
